# The Plasminogen Activation System in the Central Nervous System: Implications for Epilepsy and Neuropsychiatric Disorders

**DOI:** 10.3390/ijms262210893

**Published:** 2025-11-10

**Authors:** Elena Suleymanova, Anna Karan

**Affiliations:** Department of Molecular Neurobiology, Institute of Higher Nervous Activity and Neurophysiology, Russian Academy of Sciences, Butlerova 5A, Moscow 117485, Russia; akartar.n@gmail.com

**Keywords:** plasminogen activator, neuroserpin, PAI-1, epilepsy, comorbidities, depression, anxiety, PTSD

## Abstract

Epilepsy is one of the most prevalent neurological disorders, severely impacting quality of life. The burden of epilepsy is exacerbated by high rates of neuropsychiatric comorbidities such as depression, anxiety, and post-traumatic stress disorder. The molecular mechanisms linking epilepsy to these comorbidities remain unclear. Epileptogenesis and recurrent seizures implicate multiple processes including changes in the extracellular matrix, structural and functional neuroplasticity, neuroinflammation, and neurodegeneration. The plasminogen activation (PA) system—a complex system of proteins that function as both proteases and signaling molecules—modulates these processes in the central nervous system (CNS) under normal conditions and following potentially epileptogenic insults. Notably, the PA system is also dysregulated in stress-related psychiatric disorders. In this review, we first provide an overview of the role of PA system in the CNS with an emphasis on the mechanisms related to epilepsy. We then explore the hypothesis that the components of the PA system components constitute a shared pathological link implicated in both epileptogenesis and psychiatric disorders. We summarize clinical and preclinical evidence demonstrating that seizures and other brain insults disrupt the PA system, and that similar dysregulation is observed in stress-related psychiatric conditions. We propose that PA system dysregulation is a potential molecular substrate linking epileptogenesis and neuropsychiatric comorbidities, presenting a promising target for future research aimed at understanding the mechanisms underlying the development of behavioral comorbidities in epilepsy.

## 1. Introduction

As one of the most common neurological disorders, epilepsy impacts large global populations. The number of patients with epilepsy is estimated to be approximately 50 million worldwide [1]. This burden is frequently exacerbated by comorbid disorders—conditions that co-occur with the primary disease. In the case of epilepsy, approximately 50% of patients with active epilepsy have at least one comorbid disorder [2]. Neuropsychiatric disorders, including anxiety, depression, and post-traumatic stress disorder (PTSD), are often reported in epilepsy patients. These disorders worsen the patient’s condition, reduce quality of life, and can interfere with treatment compliance [3]. Neuropsychiatric disorders are very common in epilepsy patients with an incidence over 2–3 times higher than in the general population [4]. Among them, anxiety is reported as the most frequent, particularly in patients with intractable temporal lobe epilepsy (TLE) [5], while depressive disorders are the most extensively studied and are often associated with pharmacoresistance and poor seizure control [6]. The reported prevalence of PTSD varies but is consistently suggested to be high [7]. The significant impact of these comorbidities on patient prognosis drives research of their mechanisms and the search for new therapeutic targets. 

The molecular mechanisms underlying neuropsychiatric comorbidities in epilepsy patients remain unclear despite the notable prevalence of these comorbidities. This is particularly true for anxiety and PTSD, which have attracted less research attention compared to depressive disorders. 

The plasminogen activation (PA) system refers to a group of proteins involved in the regulation of the conversion of the zymogen plasminogen to the active serine protease plasmin, which plays a key role in fibrinolysis [8,9]. While initially studied in the context of hemostasis and thrombolysis, the PA system is now recognized as a potent modulator of key processes in the brain. Its components—proteases and protease inhibitors—are involved in synaptic plasticity, long-term potentiation, neurite outgrowth, blood–brain barrier integrity, and neuroinflammation under both physiological and pathological conditions [10,11,12]. Importantly, synaptic remodeling and neuroinflammation directly influence neuronal excitability and provide the substrate for epileptogenesis [13], suggesting that the PA system components can contribute to the pathogenesis of epilepsy by the regulation of these processes. At the same time, accumulating experimental and clinical data suggest that the PA system may be involved in the pathogenesis of anxiety, depression, and PTSD [14,15,16]. This converging evidence positions the PA system as a potential mechanistic link between epilepsy and its neuropsychiatric comorbidities.

In this review, we provide a brief overview of the PA system functions in the brain. We summarize findings from clinical and experimental studies on how seizure activity dysregulates this system and how its components contribute to key epileptogenic processes. We also explore the evidence linking PA system dysfunction to depression, anxiety, and PTSD, which are common comorbidities in epilepsy patients.

## 2. The Plasminogen Activation System and Its Function in the Central Nervous System

The PA system is a key regulator of numerous processes in the CNS. To understand its role in epilepsy and neuropsychiatric disorders, we first provide an overview of its components and their function in the CNS. The broad-spectrum protease, plasmin, is synthesized as an inactive zymogen, plasminogen, which is converted into its active form by plasminogen activators: tissue-type plasminogen activator (tPA) and urokinase-type plasminogen activator (uPA) [17]. The PA/plasminogen proteolytic cascade is regulated by specific serine protease inhibitors (serpins), most notably plasminogen activator inhibitor-1 (PAI-1, SERPINE1) and neuroserpin (SERPINI1), which serve as the primary physiological inhibitors of tPA [18]. In addition, two other serpins that can inhibit PAs have been found in the central nervous system (CNS): PAI-2 (SERPINB2) and protease nexin-1 (PN-1, SERPINE2). Serpins are evolutionary conserved proteins and the most widespread class of proteinase inhibitors that can be found in many species [19,20]. 

**Figure 1:**
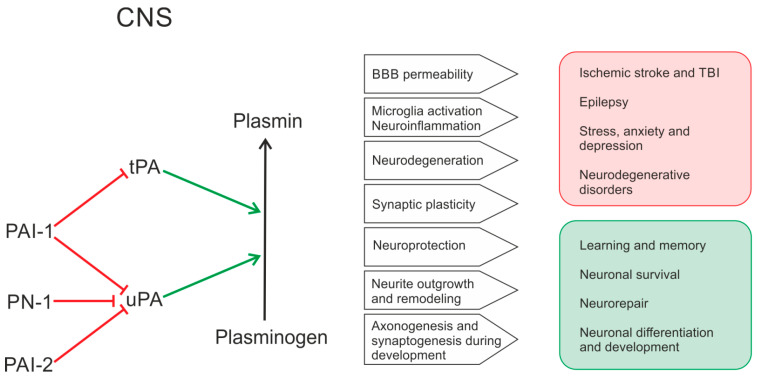
The plasminogen activation system in the central nervous system. CNS: central nervous system; tPA: tissue-type plasminogen activator; uPA: urokinase-type plasminogen activator; PAI: plasminogen activator inhibitor; PN-1: protease nexin-1; BBB: blood–brain barrier; TBI: traumatic brain injury. Red arrows indicate inhibition; green arrows indicate activation.

The PA system participates in a wide spectrum of physiological and pathological processes in the CNS. It is involved in the pathogenesis of multiple neurologic disorders, from ischemia and traumatic brain injury (TBI) to epilepsy and neuropsychiatric disorders. The PA system is also important for maintaining normal CNS activity. Figure 1 summarizes the functions of PA system in the CNS in health and disease.

### 2.1. Structure and Expression of PA System Components

#### 2.1.1. Plasminogen/Plasmin Activation by tPA and uPA

Plasminogen is expressed at low levels in brain tissues, including the hippocampus, cortex, cerebellum, and neuroendocrine tissues [21,22]. Plasminogen expression can be induced in neurons by excitotoxicity and upregulated by neurotrophins [23]. While its primary source in the CNS is transport from the systemic circulation, where it localizes to the extracellular space, plasminogen is also expressed locally within the cortex, hippocampus, and cerebellum of both immature and adult mice [22]. The conversion of plasminogen to plasmin is mediated by two serine proteases: tPA and uPA. These enzymes share a similar multi-domain structure. tPA consists of several domains: a finger domain (homologous to finger-like structures of fibronectin), epidermal growth factor-like domain (EGF), two kringle domains (similar to the kringle structures in prothrombin), and the active site of serine proteases [24,25]. Similarly, uPA is composed of a growth factor-like domain (GFD), a kringle domain, and a serine protease domain. The GFD domain contains a receptor-binding site responsible for the interaction with the uPA receptor, uPAR [26].

The expression of tPA in the CNS has been found in the microvascular endothelium of cerebral capillaries, which is considered one of the primary sources of tPA in the brain [27]. tPA expression has also been observed in several other cell types in the brain, including neurons [28,29,30] and activated microglia [31,32]. In the adult murine brain, the expression of tPA has been reported in oligodendrocytes, mastocytes, and ependymocytes [33,34]. The expression of tPA has been detected throughout the brain, but the most prominent expression has been reported in the limbic structures [30,35]. The enzymatic activity of tPA does not always correspond to its sites of synthesis; the most prominent tPA activity is found in defined areas within the hippocampus and hypothalamus [29]. Under normal conditions, the expression of uPA is practically absent in the adult brain [29]; however, under pathological conditions, such as acute ischemia and hypoxic damage, uPA can be released by neurons [36,37]. uPA is substantially more abundant during development than in the mature brain [38]. Initially uPA is secreted as a single-chain zymogen, pro-uPA, with low activity, which is activated and modified post-translationally by various proteases [39]. Binding of pro-uPA to uPAR facilitates the conversion of pro-uPA to active uPA, leading to a substantial increase in the cleavage of plasminogen to plasmin [40,41]. The receptor acts as an anchor focusing extracellular proteolysis at the cell surface [41]. The expression of uPAR has been found in microglia, endothelial cells, astrocytes, and dendritic and axonal growth cones [42,43,44]. uPA and uPAR have several functions that do not depend on proteolytic activity, when uPAR activates intracellular signaling pathways, and its ability to bind uPA is required for signaling [45]. Activation of signaling pathways via uPAR promote cell migration, tissue remodeling, cell proliferation and survival [46].

The activation of plasminogen into its active two-chain form, plasmin, is catalyzed by tPA and uPA through the cleavage of an Arg-Val peptide bond. The resulting plasmin can reciprocally cleave both tPA and uPA into their respective two-chain forms, which display distinct inhibitory properties and affinity for ligands [47,48,49]. Consequently, a bidirectional relationship exists among tPA, uPA, and the plasminogen/plasmin system, creating a complex network of mutual activation and regulation [50].

#### 2.1.2. The Inhibitors of Plasminogen Activators

The activity of tPA and uPA is regulated by a number of inhibitors. PAI-1 is a primary regulator of the activity of both tPA and uPA [51]. PAI-1 is a member of the serpin superfamily with a primary structure consisting of a single domain with an exposed loop called the reactive center loop (RCL), which is critical for its proteinase inhibitor function [52]. The RCL contains a “bait” peptide bond, which imitates the substrate of target proteases [53]. In the brain, expression of PAI-1 has been found in astrocytes and neurons in both rodents and humans [54]. Studies on neuronal/astrocyte co-cultures have shown that astrocytes are the predominant source of PAI-1 expression [55]. Under the normal conditions, PAI-1 expression in the CNS is low, but it rapidly increases after brain insults such as ischemia and inflammation [56,57]. 

Unlike PAI-1, which is abundantly expressed in the peripheral tissues, neuroserpin is a serpin predominantly expressed in neurons [58,59]. Neuroserpin shares the classical conformation of inhibitory serpins—a core of three beta-sheets surrounded by a number of helices. It also contains a flexible RCL, which is essential for its inhibitory function [60]. Neuroserpin is expressed in the neurons of both developing and mature brains [58]. The most prominent expression of neuroserpin mRNA in the murine brain was found in the neocortex, the hippocampus, the olfactory bulb, and the amygdala [61]. In humans, neuroserpin is also predominantly expressed within the central nervous system, with the most prominent presence in the frontal and temporal lobes, putamen, occipital pole, medulla, and spinal cord. Importantly, this serpin is found in many of the same regions that also express tPA [59]. 

Neuroserpin’s primary inhibition target appears to be tPA, while the inhibition rate of uPA is approximately 25-fold slower than that of tPA. At the same time, neuroserpin inhibits tPA approximately 30 times slower than PAI-1 [59]. Neuroserpin apparently carries out the regulation of basal levels of tPA in the neural tissues, unlike PAI-1, whose rapid upregulation occurs in response to brain insults. Nevertheless, the upregulation of neuroserpin is also observed after damaging events such as cerebral ischemia, when neuroserpin protects the brain tissues by inhibiting tPA and blocking tPA-induced neurodegeneration [62].

In addition to PAI-1 and neuroserpin, other serine protease inhibitors can be involved in the regulation of the PA system function. One of them is PAI-2, which belongs to the ovalbumin branch of serpins [63]. PAI-2 is an efficient inhibitor of uPA and the two-chain form of tPA, though it acts much slower than PAI-1. Its poor inhibition of single-chain tPA, especially compared to PAI-1, makes its physiological role in inhibiting single-chain tPA in vivo unlikely [64,65]. In the CNS, expression of PAI-2 has been found in microglia in the human brain; activated microglia demonstrates higher levels of PAI-2 expression. It is particularly high in microglial aggregates associated with senile plaques in brain sections from Alzheimer’s disease patients [66]. In human autopsy brain tissue with severe brain injuries, PAI-2 immunoreactivity is increased in astrocytes, microglia and endothelial cells around the lesion zone [67]. 

Another member of the serine protease inhibitors family is PN-1, which was originally purified from cultured glioblastoma cells and found to be a neurite outgrowth promoting factor [68]. PN-1 is a potent serine protease inhibitor that forms a complex with proteases such as uPA, tPA, thrombin, and trypsin with the highest affinity to thrombin [69,70].

While PN-1 is able to inhibit a few serine proteases such as uPA, the neuroprotective properties of PN-1 described in a few models of neuronal injury are typically associated with the ability of PN-1 to inhibit thrombin. An increase in PN-1 in neurons is induced by pro-inflammatory cytokines [71]. In cultured human astrocytes, an increase in PN-1 was found along with PAI-1 upregulation in response to injury-related factors such as pro-inflammatory cytokines [72].

To summarize, the activity of the PA system is under the complex control of a number of inhibitors with distinct properties. PAI-1 serves as the major systemic, inflammation-responsive inhibitor for both tPA and uPA. In the CNS, neuroserpin provides a brain-specific, fine-tuning mechanism for tPA that is vital for neuroprotection and plasticity, while PAI-2 acts as an intracellular regulator focused on uPA, with implications for microglial function in chronic pathology, and PN-1 regulates thrombin-mediated neuroinflammation and neurotoxicity. 

As the overview of the PA system components demonstrates, the PA system is very complex with components expressed at different cell types in the brain. The summary of the PA component localization and functions in the CNS is shown in Table 1. role of the PA system and its underlying mechanisms are discussed in more detail below.

### 2.2. Functional Roles and Molecular Pathways of the PA System in the CNS

#### 2.2.1. The PA System and Extracellular Matrix, Blood–Brain Barrier, Neuroinflammation, and Neurodegeneration

One of the most established functions of the PA system, along with fibrinolysis, is the regulation of extracellular matrix (ECM) metabolism. ECM is a complex structure consisting of multiple structural and non-structural proteins that function as a supporting environment for neurons, glia, and endothelial cells, and also participate in cell-surface and cell–cell interactions, regulation of cell signaling, and ultimately provide an active environment for cell differentiation, migration, axonal growth and repair, and synaptic plasticity [73,74].

Plasminogen together with tPA and uPA can bind to ECM proteins, such as laminin and fibronectin, and activated plasmin degrades ECM components [75,76,77], thus directly participating in ECM remodeling. Another ECM regulation pathway includes activation of matrix metalloproteinases (MPPs) by plasmin [78,79]. MMPs are proteolytic proteins critically involved in the ECM-related processes that regulate the structural reorganization of synapses and physiological aspects of synaptic plasticity, such as the functioning of receptors and ion channels and LTP [80,81]. MMPs are synthetized as inactive proMMPs containing a pro-peptide domain with a “cysteine switch” sequence, which suppresses their activity [82]. Activation of proMMPs occurs via proteolytic cleavage into the active form by a number of extracellular proteases, including plasmin [82]. 

An important role in the regulation of pericellular modifications of ECM belongs to the uPA/uPAR system. As mentioned above, uPAR binds pro-uPA and uPA and localizes them to the cell surface via a GPI anchor. Activated uPA converts plasminogen into plasmin, which in turn reciprocally activates pro-uPA, creating a feedback loop for localized proteolysis [46]. 

Regulation of ECM structure and function is tightly interconnected with the control of the blood–brain barrier (BBB) permeability and neuroinflammatory responses. The BBB is a selective barrier that maintains cerebrovascular homeostasis by tightly controlling the transport of molecules between the blood and the CNS. It is primarily composed of specialized brain endothelial cells, which are interconnected by tight junctions, and also includes astrocytes and pericytes [83,84]. BBB disruption is a critical event in the pathogenesis of a number of neurological conditions such as ischemic damage, neurodegenerative disorders (multiple sclerosis and Alzheimer’s disease), and epilepsy [85]. Studies on the effects of endogenous and recombinant tPA on the BBB suggest that tPA actively participates in the modulation of BBB permeability by activating MMPs, which degrade tight junctions in the BBB, leading to its damage and increased permeability [86]. Notably, evidence indicates that the interaction between tPA and PAI-1, leading to the formation of tPA:PAI-1 complexes, increases MMP-3 expression and disrupts the BBB following traumatic brain injury (TBI) [87]. This finding demonstrates that PA inhibitors can play a complex role in modulating brain damage after various brain insults.

BBB breakdown under pathologic conditions can be exacerbated by the transport of vascular tPA to the brain tissues mediated by the lipoprotein receptor-related protein 1 (LRP1) on endothelial cells [88], and inhibition of this pathway has been found to minimize the brain damage induced by ischemic stroke [89].

tPA can induce neurotoxicity by binding NMDA receptors and affecting NMDA receptor signaling, which leads to enhanced calcium influx and neurotoxicity [90]. Modulation of NMDA receptors by tPA via several pathways is reported, including plasmin-dependent cleavage of the receptor and activation of the ERK(1/2) pathway [90,91]. Since endothelial NMDA receptors participate in the BBB modulation [92], this is also a possible pathway by which tPA affects BBB function. 

The components of the PA system regulate proteolytic modifications of ECM and alter BBB permeability, and they also bind various ligands and receptors activating intracellular signaling pathways. Plasminogen binding to the plasminogen receptor Plg-RKT plays a critical role in the recruitment of leukocytes and macrophages into the inflammatory site [93]. It has been demonstrated that plasminogen is a key mediator regulating the migration of macrophages from the perivascular space into the brain during inflammation, which suggests the importance of plasminogen in communication between peripheral and central immune responses [94]. Plasminogen also can induce microglia activation and expression of pro-inflammatory mediators including interleukin IL-1β, tumor necrosis factor TNF-α, and inducible nitric oxide synthase iNOS [95]. 

tPA participates in the activation of microglia in response to excitotoxicity and ischemic damage [31,32,96]. Activation of microglia by tPA is plasminogen-independent, and tPA acts as a cytokine, binding various membrane proteins. One of these proteins is microglial LRP1. It has been demonstrated that under the ischemic conditions, tPA binding to LRP1 facilitates the expression of iNOS by activated microglia and an increase in NO production [96]. 

Another pathway of tPA-mediated regulation of microglia includes binding annexin A2 and galectin-1 membrane proteins and activating ERK1/2, PKB/Akt, and JNK signaling pathways [97]. Activation of galectin-1 has been shown to negatively regulate microglia activation and reduce microglia-mediated excitotoxic neuronal death via regulation of p38 MAPK, CREB, and NF-κB signaling pathways [98], while tPA binding to annexin A2 activates microglia via the NF-κB pathway and promotes the production of pro-inflammatory cytokines and cell apoptosis [99,100].

Studies on knockout uPA−/− and uPAR−/− mice showed the involvement of uPA/uPAR in the regulation of CNS inflammation: uPA/uPAR deficiency led to more severe manifestation of experimental autoimmune encephalomyelitis with higher levels of microglia activation and exacerbated neuronal injury and death [101]. Various insults inducing neuroinflammation and neuronal death, such as lipopolysaccharide (LPS) and KA administration significantly increased both mRNA and protein expression of uPAR in the murine hippocampus [42].

#### 2.2.2. PA Inhibitors in Regulating Neuroinflammation and Neurotoxicity

The rapid upregulation of PA inhibitors following brain damage appears to form a negative feedback loop, protecting the brain from excessive neuroinflammation and neurotoxicity. Under pathological conditions such as ischemia, neuroserpin can affect the severity of brain damage and regulate neuronal survival. An increase in neuroserpin expression induced by ischemic damage protects the brain tissues by inhibiting tPA and blocking tPA-induced neurodegeneration [62]; however, it has been reported that the neuroprotective properties of neuroserpin can also be mediated via tPA-independent mechanisms [102]. PAI-1 can have a protective role, as demonstrated by reduced lesion volume when PAI-1 is overexpressed or administered in a middle cerebral artery ligation model [103]. PAI-1 upregulation occurs after tPA expression reaches its peak, which attenuates tPA-induced neuronal and microglial toxicity [104]. Conversely, PAI-1 can exacerbate injury in a thrombosis model, where it increased the damage volume [103]. In ischemia–reperfusion injury, PAI-1 promoted the accumulation of neutrophils in the reperfused tissue, which played an important role in the initiation of the post-ischemic inflammatory response [105]. Some studies on PAI-1 deficient mice have shown that PAI-1 deficiency leads to attenuation of the inflammatory response, microvascular dysfunction, and tissue injury in experimental stroke [105,106]. 

PAI-2 deficient mice do not exhibit an increase in ischemic brain lesions, which suggests that PAI-2 is not critical for protection against ischemic damage [107]. In a traumatic brain injury model, PAI-2, however, contributes to the formation of brain edema [107]. The cytoprotective properties of PAI-2 have been shown in murine embryonic fibroblasts exposed to proteotoxic stress associated with protein misfolding and proteostasis dysfunction [108].

The protective effects of PN-1 against neuronal injury, achieved by inhibiting thrombin, and the upregulation of PN-1 in response to pro-inflammatory factors suggest that PN-1 could be involved in brain protection from trauma. This role is further supported by findings in models of experimental ischemia, where the absence of PN-1 in knockout mice exacerbates neuronal death, likely due to unregulated thrombin activity [72]. The significance of this thrombin-inhibiting function is underscored by the fact that thrombin and prothrombin can activate microglia and induce the release of pro-inflammatory factors [109,110]. Therefore, PN-1 may protect the brain by disrupting a critical inflammatory cycle: it inhibits thrombin, which in turn attenuates thrombin-driven microglial activation and the subsequent release of damaging inflammatory mediators.

#### 2.2.3. PA System in Neuronal Plasticity, Growth, and Repair

Evidence for tPA’s role in synaptic plasticity and long-term potentiation (LTP) emerged from its identification as an immediate-early gene rapidly upregulated by neuronal activity [111]. This role was further supported by behavioral studies: learning a complex motor task dramatically increased tPA expression in cerebellar granule neurons, while inhibiting tPA pharmacologically impaired the acquisition of new motor memory [112]. 

Several mechanisms of tPA participation in the formation of LTP have been proposed. The receptor LRP1 abundantly expressed in hippocampal neurons binds tPA and activates the cAMP protein kinase pathway contributing to the formation of LTP [113]. 

tPA/plasmin-induced cleavage of the brain-derived neurotrophic factor (BDNF) has been demonstrated to play a crucial role in long-term hippocampal plasticity [114]. BDNF is a neurotrophin, which effects are mediated by binding to the TrkB receptor and activating the TrkB signaling pathway, thereby regulating processes vital for brain development and function, including neuronal differentiation, adult hippocampal neurogenesis, and synaptic plasticity [115,116,117]. The transformation of the precursor form of the brain-derived neurotrophic factor proBDNF into the mature BDNF (mBDNF) is critically regulated by the PA system: tPA-generated plasmin directly cleaves proBDNF [115]. This conversion is not just the activation of a precursor form of the protein, as proBDNF and mBDNF are both functional and bind to distinct receptors, mediating opposing biological effects [118]. The ratio of proBDNF to mBDNF is important for the modulation of dendritic spine structure in different contexts and in the regulation of apoptotic pathways acting as a switch between neuronal death and survival [119,120].

The PA system’s non-proteolytic functions, which depend on receptor binding and activation of cellular signaling pathways, play an important role in neuronal survival and repair after injury. tPA participates in these processes by activating the LRP1 receptor. The binding of ligands like tPA to LRP1 triggers a signaling cascade: it activates Trk receptors through Src kinases [121,122] and subsequently kinases such as ERK1/2, which promote neuronal survival and neurite outgrowth [123]. Activation of uPA/uPAR signaling also plays an important role in the protective and regenerative processes in response to injury. Following axonal damage, an increase in uPAR expression in the growth cones of regenerating axons and subsequent enhanced binding of uPA to uPAR promoted axonal regeneration thus contributing to CNS recovery after injury. This process is mediated by LRP1 receptors, which induce membrane recruitment of active β1 integrin leading to the activation of pathways promoting axonal repair [124].

#### 2.2.4. PA System and Cell Adhesion and Migration

The PA system is involved in the regulation of cell adhesion and migration via uPA/uPAR and PAI-1 signaling. These processes are crucial during CNS development and also participate in the pathogenesis of several pathological conditions, such as tumor progression and disorders associated with abnormal neovascularization [125].

As mentioned above, uPAR signaling involves a number of proteins, including integrins and vitronectin. Vitronectin is a blood protein that is also present in the ECM [126,127]. The interaction between uPAR and vitronectin has been found to be critical for changes in cell morphology, adhesion, and migration [128]. Notably, uPA binding to uPAR causes allosteric changes in the uPAR structure and stabilizes the vitronectin-uPAR binding [129], which makes uPA an important factor in the vitronectin-uPAR signaling. 

Another important member of the signaling pathway regulating cytoskeletal changes and cell migration is integrins. Integrins are a family of heterodimeric proteins consisting of α- and β-subunits and binding to components of the ECM [130]. Recruitment of the αvβ5 integrin by uPA/uPAR promotes cell migration, and this process can be blocked by PA inhibitors [131]. Interactions of uPA/uPAR with integrins activate intracellular kinase pathways and modify processes related to cell migration [132]. PAI-1 proteolytically regulates uPA activity; however, PAI-1 signaling is also involved the regulation of cell migration via the formation of complexes with uPA/uPAR and LRP1, which leads to internalization and degradation of uPA/uPAR [133].

**Figure 2:**
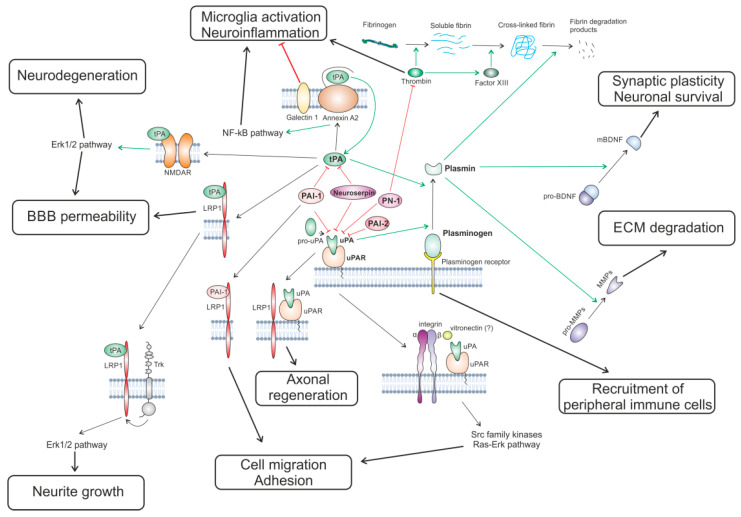
A simplified scheme of the PA system proteolytic and signaling pathways in the CNS. BBB: blood–brain barrier; Erk: extracellular signal-regulated kinase; LRP1: lipoprotein receptor-related protein 1; MMP: metalloproteinase; NF-kB: the nuclear factor kB; PAI: plasminogen activator inhibitor; PN-1: protease nexin-1; Trk: tropomyosin receptor kinase; tPA: tissue-type plasminogen activator; uPA: urokinase-type plasminogen activator; uPAR: urokinase-type plasminogen activator receptor. Red arrows indicate inhibition; green arrows indicate activation.

In summary, the PA system is involved in the regulation of a wide variety of processes in the CNS via its proteolytic function and a complex net of interactions with ECM and transmembrane proteins. PA system components comprise a complex system of proteases that interact in a tightly regulated manner and play a crucial role in extracellular proteolysis, which is involved in the regulation of normal ECM functions and neuronal plasticity and in the response to brain insults resulting in BBB damage and neuroinflammation. In addition to their proteolytic functions, PA system components can act as signaling molecules via binding to a number of receptors such as LRP1, integrins, NMDA receptors on the membranes of neurons, microglia, and endothelial cells. This receptor binding activates intracellular kinase cascades promoting the processes of neurorepair, cell migration, and synaptic plasticity. A simplified diagram of the mechanisms of PA system functions in the CNS is shown in Figure 2.

## 3. Plasminogen Activation System in Epilepsy

Epilepsy encompasses a diverse spectrum of disorders characterized by an imbalance between neuronal excitation and inhibition that results in hyperexcitability and the generation of seizures. These disorders have various etiologies. In many cases the cause of epilepsy is unknown, but in acquired epilepsies, chronic seizures develop as a result of a triggering event. Such insults include stroke, traumatic brain injury, infectious and autoimmune diseases, and genetic mutations [134]. The initial insult can trigger a cascade of events that eventually culminates in the occurrence of spontaneous seizures, a process known as epileptogenesis [13]. 

Studies on rodent models of seizures and epileptogenesis that reproduce features of human epilepsy have provided data on pathogenic mechanisms and helped identify therapeutic targets [135,136]. The broad spectrum of cellular and molecular processes involved includes neurodegeneration and neurogenesis, changes in the function of neuroglia, neurons, and the cerebral vascular system leading to BBB damage, neuroinflammation, axonal sprouting and damage, and alterations in the extracellular matrix [13,134,137]. At the same time, an epileptic seizure can itself act as a trigger, causing glutamatergic toxicity and inducing a cascade of events leading to further progression of neuronal excitability [138]. The PA system is involved in the regulation of many of these processes in the brain. A triggering event can induce profound changes in the expression of the components of the PA system.

### 3.1. PA System in Epilepsy: Experimental Animal Models 

Dramatic changes in the expression and activity of the components of the PA system in response to seizure activity are well documented. Qian et al. showed that acute seizures induced by chemoconvulsant pentylenetetrazol (PTZ) caused rapid upregulation of tPA mRNA in the hippocampus. Their work demonstrated that the gene encoding tPA could be considered as an immediate-early gene due to its rapid upregulation within 1 h after seizures [111].

A rapid increase in tPA within hours after seizures was also found in the kainic acid (KA)-induced seizure model. Administration of KA, which activates KA receptors of glutamate and induces epileptic activity, led to an increase in tPA protein levels and elevation of tPA activity in the hippocampus within a few hours after intracerebral KA injection with a peak at 4 h [139]. An early increase in tPA mRNA expression in the hippocampus and cortex was also found in the pentylenetetrazol seizure model in rats and mice [140,141] and in the amygdala kindling model [142]. 

Significant insights on the role of tPA in regulation of seizure activity and modulation of seizure-induced processes were obtained from experiments on mice with tPA deficiency. It was shown that homozygous tPA−/− mice were resistant to KA-induced seizures and neurotoxicity in comparison to wild-type controls [10]. At the same time, studies showed that plasminogen deficient mice did not have altered susceptibility to KA-induced seizures unlike tPA-deficient mice, which could be the evidence of plasminogen-independent role of tPA [143]. 

While studies on acute seizure models have demonstrated that epileptic activity induces rapid upregulation of tPA, in the case of chemically and electrically induced seizures it is difficult to distinguish the effect of seizures from the damaging effect of seizure-induced agent. In the experimental models reproducing human acquired epilepsy, the development of spontaneous seizure activity occurs over a few weeks or months after the brain damage, when epileptogenesis takes place during the seizure-free latent period [136]. These models, along with kindling models in which epileptogenesis is induced by repeated subthreshold stimulation of epileptogenic brain structures [144], are particularly useful for evaluating the changes in the PA system occurring during epileptogenesis, which is potentially relevant to the changes occurring in human acquired epilepsies. 

Studies on the model of chronic epilepsy induced by KA status epilepticus (SE) have shown that tPA participates in the regulation of mossy fiber reorganization induced by seizure activity: tPA deficient mice were characterized by decreased mossy fiber outgrowth and sprouting in the dentate gyrus, while mossy fiber outgrowth was not altered in plasminogen-deficient mice, demonstrating that tPA mediates mossy fiber reorganization, at least partially, via a plasminogen-independent mechanism [145]. tPA upregulation was also observed after repeated electric stimulation of the perforant path in an electric kindling model reproducing changes in neuroplasticity during epileptogenesis [111]. 

Despite the fact that a number of studies have shown tPA upregulation in response to seizures and its participation in the remodeling processes in the brain structures, tPA itself apparently is not directly involved in epileptogenesis. It has been shown that tPA overexpression can lower the threshold for electrically induced seizures, although tPA−/− mice were not different from the wild type in this regard. Both overexpressing tPA and tPA−/− mice were not different from wild type in the development of amygdala kindling, indicating that neither overexpression nor lack of tPA affected epileptogenesis in the amygdala kindling model [142]. At the same time, expression of tPA and other components of the PA system has been reported to change during epileptogenesis in models of acquired epilepsy induced by status epilepticus (SE). Upregulation of tPA gene was found in the rat hippocampus in the lithium-pilocarpine model of SE, and this upregulation was observed up to 3 days after the initiation of seizures [146]. Studies on transcriptomic changes in a post-SE model of epileptogenesis have shown increased expression of tPA and uPA genes during the acute and latent phases after SE, when epileptogenesis occurrs [147,148]. Long-term tPA and uPA upregulation has been observed after seizures induced by acute organophosphate intoxication. The increase in tPA and uPA persisted up to 28 days after the exposure to organophosphate-induced seizures [149], however it is unclear if those changes could be related to epileptogenesis. Our recent findings in the lithium-pilocarpine model of SE suggest that SE trigger persistent changes in the mRNA expression of PA system components in the limbic structures, which are observed in the latent and chronic period after SE [150].

Seizure activity was found to also induce an increase in neuroserpin expression in the limbic system, while administration of exogenous neuroserpin delayed the propagation of seizure activity and inhibited KA-induced tPA upregulation [143]. At the same time, neuroserpin deficient Nsp−/− mice demonstrated a phenotype opposite to that of tPA−/− mice, with significantly more severe seizures and decreased latency to seizure onset [151].

An upregulation of PAI-1, similar to that of tPA, has been reported in several seizure models. An increase in PAI-1 mRNA expression was observed in the limbic structures and cortex following KA-induced seizures in mice [11], and in the anterior cortex after pentylenetetrazol-induced seizures [140]. In the pilocarpine-induced status epilepticus (SE) model, PAI-1 protein levels in the hippocampus increased during the acute period after SE and remained elevated for at least three days [152]. Similar transient increase in PAI-1 expression was observed after organophosphate intoxication-induced seizures, which resolved by day 7 after the exposure to the toxin [149]. Interestingly, inhibition of PAI-1 attenuated the SE-induced increase in proBDNF levels, demonstrating that the PAI-1 upregulation triggered by a damaging event can promote proBDNF accumulation by inhibiting its cleavage [152]. 

Substantial data demonstrate that seizures can alter uPA/uPAR expression in the brain, and these proteins can be involved in the pathophysiological processes associated with epileptic activity. Upregulation of uPA was found in the limbic structures and cortex of mice [11]. During epileptogenesis induced by SE, an upregulation of uPA gene, as well as increase in uPA protein level and enzyme activity was found in the rat hippocampus [153,154]. uPAR expression was dramatically increased in parvalbumin positive interneurons in the hippocampus and dentate gyrus, and in a subgroup of somatostatin and neuropeptide Y positive hilar interneurons during epileptogenesis in a post-SE model [155]. A sustained increase in uPAR mRNA expression has been found in the entorhinal cortex in the latent and chronic period in the lithium-pilocarpine model [150].

Studies on knockout mice with uPA and uPAR deficiency have shown that uPAR is possibly involved in seizure susceptibility and epileptogenesis. It was reported that 5.9% of uPAR−/− mice demonstrated spontaneous seizure activity while wild type mice did not exhibit any; they also had a significantly increased susceptibility to the chemoconvulsant pentylenetetrazol compared to wild types [155]. Further studies on intrahippocampal KA epilepsy model in knockout uPAR−/− mice showed that while mice with uPAR deficiency did not demonstrate higher sensitivity to KA in comparison to wild types, they had higher severity of spontaneous seizures and more severe hippocampal neurodegeneration [156].

At the same time, post-TBI epileptogenesis was not different in uPAR deficient mice in comparison with wild type mice; however, uPAR deficiency led to exacerbation of learning impairments after TBI [157]. Mice with double uPA and uPAR deficiency, demonstrated higher susceptibility to PTZ-induced seizures [158]. 

In contrast to uPAR deficient mice, uPA−/− mice did not demonstrate increased susceptibility to seizures and were not prone to spontaneous epileptiform activity [159].

While tPA and PAI-1 have been extensively studied in various models of brain disorders including epilepsy, much less is known about the role of other members of the PA system in epileptic activity and processes triggered by seizures. Nevertheless, there is evidence that seizures alter the expression of PA inhibitors other than PAI-1 as well. PAI-2 has been found to have protective effects against cytotoxicity [160], but its function in the CNS is still poorly understood. Some studies have proposed a role of PAI-2 in neuroprotective mechanisms under conditions of ischemic stroke, where intranasal administration of recombinant PAI-2 reduced the size of the ischemic lesion [161]. Rapid (within 2 h) upregulation of PAI-2 mRNA has been detected in the KA-treated mouse brain in the neocortex, the cingulate, piriform, entorhinal, and perirhinal cortices, the olfactory bulb, striatum, amygdala, and hippocampus [162].

Upregulation of PN-1 is associated with various types of brain trauma including traumatic brain injury. Brain trauma and ischemic damage induce an increase in the expression of thrombin [163,164]. Thrombin is reported to have a pro-convulsive effect, and a few studies suggested that it is one of the key factors in the development of post-traumatic epilepsy [165,166,167]. It was shown that thrombin inhibitors can block thrombin-induced seizure activity [165]. At the same time, PN-1 is a serpin with high affinity for thrombin and can promote neuronal survival [168]. However, the role of PN-1 in post-traumatic epileptogenesis remains unclear. 

Results of the studies of the components of the PA system in experimental animal models of seizure activity are summarized in Table 2.

The overview of the studies of the PA system in experimental epilepsy shows that a significant amount of data on its changes has been obtained from acute seizure models and from post-SE or post-TBI models of acquired epilepsy. It is possible that in genetic absence models, the PA system is not altered, as these models lack the massive neurodegeneration and synaptic reorganization seen in other types. However, findings from Wag/Rij and GAERS rats indicate a role for neuroinflammation in absence seizures [170,171]. Since the PA system is involved in neuroinflammation, it could also be dysregulated in these models, but direct evidence for this is lacking.

### 3.2. PA System in Epilepsy: Clinical Studies

Evidence for the involvement of PA activation system in various neurologic disorders was first provided by measuring levels of PA system components in the cerebrospinal fluid (CSF) of patients with various neurologic disorders. An increase in PAI-1 level was reported in the CSF of patients with dementia and Alzheimer’s disease, ischemic stroke, encephalitis, multiple sclerosis, and alcohol withdrawal seizures [172,173]. Plasma levels of PAI-1 were increased in patients with Alzheimer’s disease [174], ischemic stroke [175], traumatic brain injury [176]. In epilepsy patients, an increase in blood plasma levels of PAI-1 was associated with resistance to antiepileptic drugs in children [177]. 

The investigation of surgically resected brain tissue from patients with focal chronic refractory epilepsy revealed increased protein levels of tPA, uPA, and uPAR, alongside elevated mRNA expression of uPA, uPAR, and PAI-1, in the hippocampus of patients with hippocampal sclerosis [178]. In another study of human surgical tissues from patients with intractable frontal lobe epilepsy, elevated neuronal uPAR expression was found in the frontal lobe [179]. Elevated plasma levels of uPAR have been reported in TLE patients with hippocampal sclerosis, and amygdalo-hippocampectomy, which removed the epileptogenic tissue, returned uPAR levels to those of controls [180]. These findings corroborate the findings from animal models of seizures and show that activation of the PA system and upregulation of uPAR receptors occur in human plasma and epileptic brain tissues. 

The study of genomic DNA of TLE patients suggested the existence of an association between polymorphisms in the tPA gene (*PLAT*) and the development of TLE: there were significant differences in genotypic and allelic frequencies of polymorphic sites between TLE patients and controls. However, no differences in PAI-1 (*SERPINE1*) polymorphisms were found at the same time [181].

Interestingly, little is reported about tPA levels in epilepsy patients. An increase in serum tPA levels has been found in children with idiopathic epilepsy, and tPA levels have been reported to be positively correlated with the epilepsy severity [182].

tPA is widely used in clinical practice for the therapy of ischemic stroke as a thrombolytic agent. This therapeutic application has raised the question of whether the administration of pharmacological tPA (alteplase) increase the risk of post-stroke seizures [183]. Animal studies have shown that both endogenous tPA upregulated in response to the brain insult and direct intravenous and intracerebral injections of exogenous tPA exacerbate seizure activity, promote neurodegeneration, and increase the lesion size [184,185,186]. However, studies on the stroke patients who received tPA treatment showed that the incidence of post-stroke epilepsy did not depend on tPA administration [142,183,187]. Moreover, recent studies report that thrombolytic therapy with recombinant tPA decrease the risk of poststroke seizures [188,189]. While some studies reported higher rates of post-stroke epilepsy in patients undergoing thrombolytic therapy with intravenous tPA [190,191], this could be due to the factors associated with the treatment protocol and the inclusion of patients with more severe ischemic damage resulting in higher seizure incidence, rather than the epileptogenic properties of exogenous tPA [191].

Interest in neuroserpin in the context of epilepsy is mostly related not to its function as tPA inhibitor, but to the tendency of some neuroserpin variants to polymerize inside neurons, thus disrupting their function. Mutations of the *SERPINI1* gene encoding neuroserpin are associated with the development of familial encephalopathy with neuroserpin inclusion bodies (FENIB), which is often characterized by progressive myoclonic epilepsy [192]. Mutated neuroserpin is prone to progressive polymerization and formation of inclusions in neurons called Collins bodies, which eventually cause neurodegeneration and cognitive impairment [193]. It has been shown that neuroserpin containing the S49P mutation associated with FENIB is characterized by a reduced ability to inhibit proteinases and is prone to formation of loop-sheet polymers [194]. Mutations leading to the loss of tPA inhibition by neuroserpin result in the development of progressive myoclonus epilepsy as the prevalent clinical feature of FENIB [193]. 

Reported alterations in the PA system associated with epilepsy are summarized in Table 3. 

The findings from clinical studies are in line with data from experimental models and generally demonstrate the upregulation of PA system components in epilepsy patients. Notably, the changes in the PA system have been mostly reported in patients with severe forms of epilepsy resistant to antiepileptic drugs. In the case of brain tissues this could be due to the availability of the surgical material; and it is likely that changes in tPA and PAI-1 are detected only in patients with severe and frequent seizures that are accompanied by massive BBB breakdown and ongoing neurodegeneration, which is supported by the findings demonstrating the decrease in uPAR circulating level after the surgical removal of the epileptic focus [180]. Nevertheless, the association of TLE with certain polymorphisms of the tPA gene suggests a deeper connection between PA system dysfunction and epilepsy, though this requires further investigation.

### 3.3. The Mechanisms of the PA System Involvement in Seizures and Epileptogenesis

As we have outlined in the previous sections, evidence from both experimental and clinical studies indicates that the PA system is significantly altered in epilepsy. These findings suggest that the PA system constitutes a potential mechanistic link in hyperexcitability and epileptogenesis. Building on its known neurobiological functions, we further discuss specific mechanisms through which the components of the PA system may contribute to the pathogenesis of epilepsy.

#### 3.3.1. The Regulation of ECM in Epilepsy

First, as we have already discussed, the proteolytic activity of plasmin is known to alter the extracellular matrix. A number of studies have shown that the expression of ECM proteins can be significantly changed following seizures. In experimental post-SE TLE models and kindling models of epileptogenesis, seizures induced sustained alterations in the expression of ECM proteins [195,196,197,198]. Clinical findings confirm alterations in the ECM composition: studies of hippocampal tissues from TLE patients have revealed altered expression of various ECM components [199,200]. Therefore, findings from experimental models and clinical studies indicate that alterations in ECM protein composition and dysregulation of ECM remodeling can be involved in the regulation of seizure susceptibility and epileptogenesis. In the context of the regulation of synaptic plasticity and neuronal excitability, particular interest is drawn to perineuronal nets (PNNs)—specific structures within the ECM that create a support for cell bodies, dendrites, and proximal parts of axons of neurons in the cortex, hippocampus, amygdala, and some other parts of the brain and are primarily associated with parvalbumin-positive GABAergic interneurons [201,202]. The localization of PNNs around inhibitory interneurons and their role in regulating GABAergic transmission position them as important modulators of the excitation-inhibition balance. This role suggests their potential involvement in the pathogenesis of epilepsy [202]. Disruption of PNNs affects neuronal plasticity and alters neuronal excitability [203,204,205]. Recent studies have suggested that prolonged seizures during SE induce the formation of new PNNs around selected populations of neurons in the hippocampus; and these de novo PNNs contribute to the development of hyperexcitability [206]. There is evidence that tPA expressed in parvalbumin interneurons regulates PNN remodeling via activation of plasmin leading to degradation of aggrecan, one of the main PNN proteoglycans [207], which demonstrates that tPA/plasmin is potentially one of the factors regulating alterations in PNN structure during epileptogenesis.

In addition to the direct degradation of ECM components by tPA and plasmin, PA system components may also contribute to the ECM-related control of excitability and epileptogenesis by regulating the activity of MMPs. Altered MMP expression has been reported in response to acute seizures and during various stages of epileptogenesis in experimental models [146,147,148,208], as well as in epilepsy patients [180,209]. The involvement of MMPs, particularly MMP-9, in regulating neuronal plasticity and excitability is well-documented, and their role in pathological plasticity represents a potential mechanism of hyperexcitability and epileptogenesis [81,210]. However, the detailed mechanisms by which MMPs contribute to epileptogenesis, and the specific role of PA system proteases such as tPA in this process, remain to be fully elucidated and require further investigation.

#### 3.3.2. The Blood–Brain Barrier in Epilepsy

BBB damage occurs during an initial insult triggering epileptogenesis, and also seizures themselves can induce BBB leakage [211]. Altered permeability of the BBB under pathological conditions can induce ion imbalance in the brain tissues, edema, and neuroinflammation, which ultimately lead to neuronal dysfunction and neurodegeneration [85].

BBB leakage has long been observed in epilepsy patients, with transient changes appearing post- and peri-ictally on MRI [212,213,214,215]. Furthermore, it was discovered that BBB permeability changes in these patients appeared interictally, particularly in regions that were presumed to be zones of seizure onset [216]. Elevated levels of markers of BBB breakdown, such as the metalloproteinases MMP-2, MMP-9 and their inhibitors TIMP-1 and TIMP-2, were reported in epilepsy patients in the interictal period, when they did not have seizures for at least 7 days [217]. Interestingly, a study on patients undergoing BBB breakdown during chemotherapy against brain lymphomas showed that BBB disruption led to the development of acute seizures, showing that BBB breakdown itself can promote seizure susceptibility [218]. The studies of the role of BBB damage in epileptogenesis in experimental models of acquired epilepsy have also shown persistent disruption of the BBB at various stages of epileptogenesis, and the degree of BBB damage correlated with seizure frequency and could predict the severity of post-injury epilepsy [219,220]. Therefore, the BBB is apparently implicated in epilepsy at the initial stage, when its increasing permeability contributes to seizure genesis due to increased penetration of large neuroactive proteins and immune cells. Subsequently, in the chronic epileptic state, the BBB function becomes an important factor in determining the progression of the disease [221].

A few possible mechanisms for the involvement of BBB dysfunction in epileptogenesis have been proposed, such as an increase in hemorrhage, vasogenic edema, and infarct size, albumin extravasation, and neuroinflammation [222]. Opening of the BBB leads to the extravasation of serum albumin, which triggers the activation of inflammatory transforming growth factor beta (TGF-β) signaling in astrocytes and eventually leads to epileptogenesis [223]. Another possible mechanism for the contribution of BBB damage to epileptogenesis is the invasion of immune cells, including monocytes, neutrophils, and different types of T and B cells, from peripheral circulation into the brain tissues, which leads to sustained neuroinflammation and various immune cell-mediated processes exacerbating neuronal damage [224]. In the case of post-traumatic and post-stroke epilepsies, a dramatic increase in tPA expression immediately after the brain insult contributes into the opening of the BBB and the subsequent neuroinflammatory and neurotoxic events via the pathways we have previously described (Figure 1). However, there are clinical and experimental studies that do not support the direct involvement of tPA in epileptogenesis in acquired epilepsies [142,187].

#### 3.3.3. PA Activation System, Neuroinflammation, and Epilepsy

Another potential link between changes in PA activity and seizure susceptibility and epileptogenesis is neuroinflammation. Numerous studies have shown that neuroinflammation plays crucial role in the response of brain tissues to the initial insult and activation of a broad range of processes ultimately leading to epileptogenesis [225,226,227].

In human epilepsies, multiple studies on both epilepsy patients and animal models have shown the activation of inflammatory pathways in epilepsy [225,228]. Immune system pathologies in autoimmune disorders are often accompanied by the development of recurrent seizures and underlie several epileptic syndromes such as Rasmussen’s encephalitis and Lennox-Gastaut syndrome [228,229]. Increased levels of pro-inflammatory cytokines and activated microglia have been found in the brain tissues of TLE patients with hippocampal sclerosis [230,231,232].

Several neuroinflammatory pathways have been implicated in epileptogenesis. One of them is the IL-1 receptor (IL-1R1)–Toll-like receptor (TLR) 4 axis; activation of this signaling cascade contributes to neuronal hyperexcitability in animal models and is present in human pharmacoresistant epilepsy [233]. Other possible mechanisms include oxidative stress pathways, and TGFβ signaling, the latter being particularly associated with BBB dysfunction [225].

The neuroinflammatory response initiated by a brain insult is an adaptive reaction to the damaging factor. However, when endogenous resolution mechanisms fail, this response can become prolonged and maladaptive, triggering irreversible processes that contribute to chronic pathology. Such a transition from the maintenance of homeostasis to chronic pathology appears to happen in the epileptic brain [225]. The PA system is closely interconnected with neuroinflammation, as a massive rapid upregulation of PA system components, such as tPA, uPA, and their inhibitors occurs in response to a potentially epileptogenic insult. The PA system is closely involved in the regulation of neuroinflammatory response via microglia activation, BBB permeability regulation, and the regulation of infiltration of immune cells to the damaged site [21,101,184], and can be a potential link in the pathological cascade that sustains neuroinflammation.

#### 3.3.4. uPAR Signaling in Epileptogenesis

uPAR signaling is an important mechanism to consider in the pathogenesis of epilepsy. As previously outlined, altered uPAR expression is a consistent feature in both experimental models and human epileptic tissue. Furthermore, uPAR deficiency in knockout mice leads to increased susceptibility to the development of spontaneous seizures, directly implicating it in epileptogenesis [155,159]. uPAR functions as a signaling hub by forming complexes with various transmembrane receptors, including integrins, formyl peptide receptors (FPRs), and growth factor receptors like PDGFR-β [234]. These interactions regulate cell adhesion, proliferation, and migration. Disruptions in this uPAR-mediated signaling, particularly in neuronal migration, can lead to cortical malformations. These malformations, such as cortical dysplasia and lissencephaly, are well-established causes of severe, intractable epilepsy and associated seizure types [235]. While the most studied uPAR ligand, uPA, itself does not appear to have a direct pro-epileptogenic role, its binding to uPAR induces conformational changes that modulate interactions with other ligands and intracellular signaling [210].

#### 3.3.5. Synaptic Reorganization in Epileptogenesis

Studies on experimental models of acquired epilepsy have demonstrated that structural and functional neuronal plasticity is an essential part in the development of hyperexcitability and epileptogenesis [13,236]. Sprouting of granule cell axons (mossy fibers) in the hippocampus is a common finding in various models of seizures including post-SE models and kindling [237,238,239,240], and similar histopathologic findings have been reported in human TLE [241,242]. An increase in dendritic connections and axonal sprouting of inhibitory interneurons is also typical for the epileptic hippocampus [243,244]. It is hypothesized that massive synaptic reorganization creates aberrant connections and abnormal excitatory circuits. Together with the degeneration of inhibitory neurons from the initial brain insult, this shift pushes the excitation/inhibition balance toward hyperexcitability, leading to recurrent seizures [236].

BDNF/TrkB signaling plays an important role in the regulation of axonal sprouting. An increase in BDNF expression is induced by seizure activity, and elevated BDNF levels promote dendrite formation and axonal sprouting [245,246].

BDNF/TrkB is also involved in the regulation of dendritic spine density and maturation [247] and maintains the spine structure in activity-dependent manner [248]. Activity-dependent activation of BDNF signaling is crucial for the long-term maintenance of LTP [249]. The regulation of proBDNF to mBDNF by tPA/plasmin is a potential link between PA system dysregulation and synaptic reorganization during epileptogenesis.

**Figure 3:**
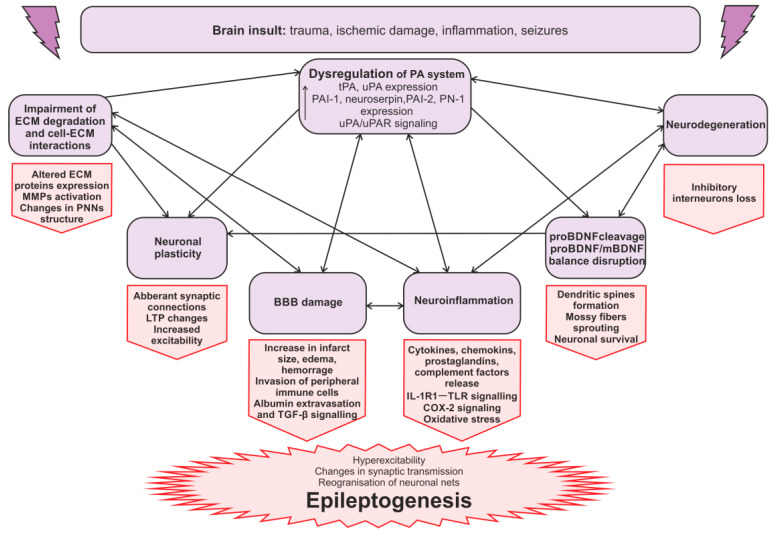
The PA system and epileptogenesis. The initial brain insult triggers a number of pathologic processes, that are mutually interconnected and create the substrate for epileptogenesis. Dysregulation of PA system is involved in the initiation and maintaining of these processes. BBB—blood–brain barrier; BDNF—brain derived neurotrophic factor; ECM—extracellular matrix; IL-1R1—interleukin -1 receptor type 1; MMP—metalloproteinase; LTP—long-term potentiation; COX-2—cyclooxygenase 2; TGF-β—transforming growth factor beta, TLR—Toll-like receptor.

Summarizing the known data on the PA system’s role in regulating BBB permeability, ECM functions, neuronal plasticity, neuroinflammation, and the mechanisms of epileptogenesis, it is evident that its components are involved in the very processes that constitute the substrate for epileptogenic changes in brain tissue. An initial brain insult induces a rapid upregulation of PA system components, which contributes to the initiation and maintenance of processes that trigger epileptogenesis. Following the development of spontaneous seizures, chronic epileptic activity further sustains these pathological processes and the associated PA system dysfunction. A schematic diagram of the proposed interactions is shown in Figure 3, illustrating how the PA system may function as a link in the multiple pathological cascades in epilepsy.

## 4. Plasminogen Activation System in Neuropsychiatric Disorders

Data from cross-sectional studies demonstrate a higher prevalence of all psychiatric disorders in adult and pediatric epilepsy populations compared to the general population [250]. Among these neuropsychiatric comorbidities, an important role belongs to mood and stress-associated disorders, such as depression, anxiety, and PTSD. While social factors, fear of recurrent seizure episodes, disability associated with seizures, and AED adverse effects play a big role in the development of neuropsychiatric disorders in epilepsy patients, epilepsy-related factors also play an important part [251]. However, the pathophysiologic mechanisms underlying the association of epilepsy with neuropsychiatric comorbidities are still not fully understood.

As has been discussed in previous sections, proteases of the PA system are involved in many processes that can potentially underlie changes in the brain associated with behavioral disorders, such as synaptic remodeling, long-term potentiation, neuroinflammation, and neurodegeneration. A substantial amount of data supports the role of the components of the PA system in the pathogenesis of depression, anxiety, and PTSD.

### 4.1. Depression

The existence of a connection between epilepsy and depression has been reported for many years. Patients with epilepsy have a substantially higher risk of depression compared to the general population, with reported prevalence rates ranging from 20% to 55% among those with recurrent seizures [252].

Multiple studies have provided evidence of the involvement of PA system components in the pathogenesis of depression. In humans, most studies investigated levels of tPA and PAI-1 in the peripheral circulation in patients with various disorders, including depression. Changes in these parameters, such as a decrease in tPA and an increase in PAI-1 levels, have been reported in patients with depression, neurosis, and chronic stress [253,254], and recent studies show that tPA and PAI-1 levels can be reliable markers of major depressive disorder (MDD). An investigation of tPA levels showed a significant decrease in serum tPA levels in MDD patients prior to therapy, while treatment with selective serotonin reuptake inhibitors reversed these changes. PAI-1 serum levels were not significantly different from controls in these studies [15,255]. In other studies, both mRNA and protein PAI-1 plasma levels were increased in MDD patients, and this increase was reversed after treatment with antidepressants [256,257]. Notably, in a study on patients with coronary heart disease associated with depression, these patients demonstrated increased tPA plasma levels, but they did not correlate with depressive mood [258].

Interestingly, while a few studies on MDD patients report increased PAI-1 plasma levels, a significant decrease in plasma PAI-1 levels has been found in patients with geriatric depression [259], indicating a more complex involvement of the PA system in the pathophysiology of depressive disorders.

While data on PA component expression in the human brain are scarce, available evidence from postmortem studies on brain tissues from patients with depression showed PAI-1 immunoreactivity in hippocampal astrocytes, with particularly prominent staining in limbic structures, which was stronger than in healthy controls [257].

The involvement of PAI-1 in MDD is supported by genetic studies linking specific polymorphisms of the *SERPINE1* gene encoding PAI-1. The rs2227684-G and rs7242-T alleles has been found at a higher frequency in MDD patients compared to healthy controls. It has been also found that certain variants correlate with a reduced therapeutic response to selective serotonin reuptake inhibitors [260].

Studies in knockout PAI-1−/− mice showed an association of PAI-1 deficiency with the development of a depressive-like phenotype with resistance to selective serotonin reuptake inhibitors. This effect of PAI-1 deficiency appeared to be independent of tPA [261]. In contrast, murine models of depression induced by stress and inflammation showed an upregulation of PAI-1. Administration of pro-inflammatory agent LPS induced depressive-like behavior in mice accompanied by an increase in plasma PAI-1 levels and increased PAI-1 expression in the hippocampus [257]. Similarly, the chronic unpredictable mild stress model of depression resulted in increased levels of PAI-1 in the prefrontal cortex and hippocampus of stressed rats [262].

Evidence implicating neuroserpin in depressive disorders is limited and inconsistent. Animal studies have reported a decrease in neuroserpin mRNA expression in the hippocampus and prefrontal cortex of rats subjected to depression models such as chronic unpredictable mild stress or LPS administration. A similar reduction was observed in the peripheral blood mononuclear cells of patients with first-episode depression [262]. Another clinical study recently reported an increase in plasma levels of neuroserpin in pregnant women diagnosed with depressive syndrome [263].

While the detailed mechanisms of the development of depressive disorders are still not fully understood, one of the hypotheses of the MDD pathogenesis involves the BDNF. It is involved in the pathogenesis of a wide spectrum of CNS disorders, including anxiety and depression [264]. Therefore, the dysregulation of the PA system is proposed as a mechanism in depression pathogenesis, potentially by altering the proBDNF/mBDNF balance. This hypothesis is supported by findings showing an increase in proBDNF and decrease in BDNF plasma levels in patients with depressive disorders [15,256].

### 4.2. Anxiety and Stress

Anxiety, like depression, has been noted as related to seizure events for a long time. Patients with epilepsy have a much higher risk of anxiety disorders than the general population. A recent meta-analysis reported that the incidence of anxiety in epilepsy patients ranged from 15.89% to 67.10% with a median incidence of 34.62% [265]. Anxiety disorders are represented by a wide spectrum of syndromes varying from generalized anxiety disorder to more specific sources of anxiety [4]. In patients with stress-related disorders, it has been long observed that cardiovascular diseases were associated with high levels of psychological stress and elevated anxiety levels [266,267]. This association was proposed to be mediated by activation of coagulation and fibrinolysis. An investigation of fibrinolytic system components in patients with anxiety disorders showed elevated plasma PAI-1 levels [268]. However, the results of another study showed no increase in either tPA or PAI-1 plasma levels in patients with panic disorder [255]. The contradictory results in clinical studies could be due to the heterogeneity of anxiety disorders and differences in the selection of patients included in the study.

Experimental studies of the relationship between the PA system and stress have shown that both tPA and its inhibitor PAI-1 are stress-induced proteins, which expression can be induced by corticosteroid dexamethasone, as well as restraint stress [269,270,271]. This regulation is facilitated by glucocorticoid-responsive elements within their gene promoters, which mediate a rapid increase in mRNA transcription [272]. Consequently, elevated levels of stress hormones directly promote the simultaneous expression of tPA and PAI-1. Despite this well-established relationship, the functional significance of tPA and PAI-1 in stress-related psychiatric disorders is not fully understood, especially when compared to their canonical role in fibrinolysis [272].

Research on the PA system’s role in stress-induced behavioral changes, much like in epilepsy, has been significantly advanced through studies on rodent models, particularly knockout mice with targeted protein deficiencies. It was demonstrated that restraint stress led to upregulation of tPA and an increase in tPA (but not uPA) activity in the amygdala. This increase was accompanied by upregulation of PAI-1, while the level of neuroserpin expression did not change in response to stress [273]. At the same time, knockout tPA−/− mice exhibited a lack of stress-induced anxiety in the elevated plus maze in response to restraint stress in contrast to wild-type mice [273]. A more recent study on the novel tPA^null^ strain of mice with reduced off-target effects have confirmed increased locomotor activity and reduced anxiety in mice with tPA deficiency. Region-specific deletion of tPA showed that tPA deficiency in the dentate gyrus led to increased locomotor hyperactivity, and tPA deletion in the central amygdala caused locomotor hyperactivity and reduced anxiety [16]. These findings from both knock-out mice and tPA removal from the mature hippocampus support evidence for the role of tPA in mediating behavioral responses to stress, moving beyond correlation to demonstrate that deficiency of the protein contributes into the development of the anxiety phenotype.

As previously discussed, it was shown that unlike PAI-1, neuroserpin is not upregulated in response to stress. Interestingly, both neuroserpin-deficient and neuroserpin-overexpressing mice have been reported to show decreased exploratory activity and neophobia [274]; however, another study has reported no changes in novelty-induced exploration and anxiety in neuroserpin-deficient mice [275].

Another member of the PA system uPA and its receptor uPAR have been shown to also be involved in the regulation of behavioral responses to stress. Although in experiments on uPA−/− mice, uPA deficiency did not lead to the development of higher anxiety levels in the elevated plus maze test, uPA-deficient mice demonstrated a reduction in exploratory activity and an enhanced fear response to tone [159]. At the same time, mice with uPAR deficiency were characterized by increased anxiety in behavioral tests. [155].

### 4.3. PTSD

Post-traumatic stress disorder (PTSD) is another comorbid neuropsychiatric disorder that is frequently reported in patients with epilepsy [276,277]. According to a recent meta-analysis, the pooled prevalence of PTSD in patients with epilepsy is 18%. These patients demonstrated a three-fold increased risk of developing PTSD compared to the general population [278].

PTSD has been reported to have an association with an increased cardiovascular risk and changes in the fibrinolysis [279], and PA system dysfunctions are known to be involved in the pathogenetic mechanisms of cardiovascular disorders. Accumulating evidence suggests that PA system components can play a role in the development of maladaptive stress-induced responses underlying PTSD [272].

Recently, Bouarab et al. showed that the shift in the tPA/PAI-1 balance is involved in the mechanisms of the formation of PTSD-like memory in mice. In this study, a systemic injection of corticosterone combined with fear conditioning caused an increase in PAI-1 expression and development of PTSD-like memory. The injection of PAI-1 into the hippocampus led to a similar effect, which could be blocked by injection of mBDNF. These results demonstrated the key role of PAI-1 in the development of PTSD, presumably mediated by tPA/plasmin conversion of proBDNF into mBDNF [14]. In transgenic VGV mice overexpressing serotonin 2C receptors in the brain and exhibiting PTSD-like behavior, tPA and BDNF mRNA expression was decreased in the hippocampus but increased in the amygdala [280]. In clinical studies, it was reported that pediatric PTSD patients had decreased serum levels of BDNF and proBDNF and increased serum tPA levels [281]. In another study on PTSD patients, increased tPA levels were also observed, while PAI-1 levels were not different between PTSD patients and control subjects [282].

Table 4 summarizes findings from studies of patients with depression, anxiety, and PTSD, as well as from animal models. Overall, human and animal studies demonstrate the involvement of the PA system in the pathogenesis of stress-related conditions, anxiety, and depression. Experimental animal studies suggest the involvement of proteolytic properties of tPA and PAI-1, as well as pathways independent of proteolysis. However, it is important to point out the limitations in interpreting the results of clinical studies, as most data were obtained from investigations of PA component levels in the blood, which may not accurately reflect changes in the CNS. In the case of depression, the involvement of the PA system is supported by genetic data and findings from post-mortem brain tissues. To date, similar confirmations for anxiety and PTSD have yet to be obtained. The inconsistency in the levels of peripheral PA system components in humans underscores the need for cautious interpretation. It remains unclear whether peripheral tPA/PAI-1 levels in anxiety and PTSD patients are related to the pathogenesis of these disorders or are the consequence of a chronic stress state and its associated systemic physiological alterations.

## 5. Prospective Role of the PA System in the Development of NeuropsyChiatric Comorbidities in Epilepsy

The PA system in the brain is a modulator of neuroinflammation, synaptic plasticity, and neuronal survival in the central nervous system. The components of the PA system are involved in the regulation of LTP and remodeling of synaptic connections, neurite outgrowth and remodeling, neuronal survival, BBB permeability, and neuroinflammation. These processes are key elements of normal brain function, and their disruption can lead to the development of hyperexcitability, ultimately leading to epileptogenesis [13,135,226]. At the same time, multiple experimental studies have shown that various brain insults, including ischemic damage and traumatic brain injury, induce rapid and profound changes in the expression of PA system components, which could play a role in a network of molecular and cellular changes underlying hyperexcitability and epileptogenesis. It is also clear from the literature that seizures themselves can induce massive changes in PA system expression and activity, maintaining an altered balance in the PA system. An overview of the changes in the PA system demonstrates that it is also intricately linked to the pathophysiology of depression, anxiety, and PTSD, which are often reported in epilepsy patients as comorbid psychiatric disorders [4,276]. Therefore, the PA system might be one of the links integrating the pathophysiology of epilepsy with the development of its behavioral comorbidities.

The PA system may act as an interconnecting link via several common mechanisms involved in the pathophysiology of brain disorders. First, the participation of the PA system in the regulation of synaptic plasticity and reorganization of neuronal connections is crucial for learning and memory formation and, at the same time, provides the substrate for maintaining pathologic synchronization during epileptic activity. Impaired learning and memory associated with altered neuroplasticity, in turn, contributes to the development of behavioral disorders [283].

Second, brain insults trigger a cascade of molecular events that induce neuroinflammation, blood–brain barrier disruption, and excitotoxicity. Activation of microglia resulting from a brain insult is accompanied by increased expression of uPAR and uPA as well as of tPA and PAI-1 [42]. Neuroinflammation has been found to be an important pathogenetic mechanism of neuropsychiatric disorders [284,285]. Consequently, the dysregulated PA system in the epileptic brain can contribute to the neuroinflammatory and neurodegenerative processes involved in the pathogenesis of mood and anxiety disorders.

Finally, there is a prospective mechanism involving BDNF signaling. The PA system activates the proteolytic cascade in which plasmin cleaves proBDNF to mature BDNF. The balance between these forms of BDNF is crucial for synaptic plasticity and neuronal survival [264]. In epilepsy, a dysregulated PA system could disrupt this balance, leading to an accumulation of proBDNF [152]. Since proBDNF and mBDNF often exert opposing effects on neuronal survival and plasticity, this imbalance may directly contribute to the hippocampal dysfunction and synaptic deficits that underpin depressive and anxiety-like behaviors [286,287]. This provides a possible molecular bridge between seizure-induced PA system changes and affective pathology.

## 6. Conclusions

In conclusion, the plasminogen activation (PA) system emerges as a critically important proteolytic network in the brain, which functions extend far beyond its classical role in fibrinolysis. It is a modulator of fundamental processes such as neuroinflammation, synaptic plasticity, and neuronal survival. Substantial evidence shows that brain insults and subsequent seizure activity induce profound and persistent dysregulation of PA system components, directly contributing to the molecular cascades that underlie hyperexcitability and epileptogenesis. The existing evidence does not yet clarify how critical the PA system is for the development of epilepsy, and this topic needs further research. However, the significance of PA system dysregulation may lie in its role in creating a chronically dysfunctional neural environment. At the same time, emerging evidence from studies in patients with mood and stress-related disorders and in animal models demonstrates the possible involvement of the PA system in the pathogenesis of such disorders. The present data on the precise mechanisms of its involvement in conditions such as anxiety, depression, and PTSD remain limited and likely vary across disorders. Research in this area faces certain limitations, including constraints of clinical studies; and methodological challenges of animal models, which only reproduce specific aspects of complex neuropsychiatric conditions. Nevertheless, the implication of the PA system in key processes underlying both epilepsy and neuropsychiatric disorders makes it a promising focus for research of the mechanisms underlying the development of behavioral comorbidities in epilepsy. Further studies focused on elucidating the specific pathways of the PA system involvement in the pathogenesis of these disorders are needed to evaluate its potential as a therapeutic target, not only for controlling seizures but also for mitigating the comorbid conditions.

## Figures and Tables

**Table 1:** The expression and functions of the PA system components in the CNS.

Component of the PA System	Expression Site	Main Function in CNS	Effect in the CNS
tPA	Endothelial cells Neurons Activated microglia Oligodendrocytes Mastocytes Ependymocytes. The most prominent expression in the limbic structures	Conversion of plasminogen to its active form, plasmin Cell signaling	Activation of microglia Synaptic remodeling Long-term potentiation formation Regulation of vascular permeability and BBB integrity Neuronal migration in developing brain Regulation of neurodegeneration and neuronal survival in various pathologic conditions
uPA/uPAR	uPA: neurons uPAR: microglia, endothelial cells, dendritic and axonal growth cones, astrocytes	Conversion of plasminogen to its active form, plasmin Cell signaling	Neuroplasticity Cell migration Cell proliferation and survival Neuroinflammation
PAI-1	Astrocytes Neurons	tPA and uPA inhibition Cell cignaling	Neuroinflammation and neurodegeneration control Cell migration
PAI-2	Microglia Astrocytes Endothelial cells	Inhibition of uPA and two-chain form of tPA	Neuroprotection
Neuroserpin	Neurons Predominantly in the neocortex, hippocampus, olfactory bulb, the amygdala	tPA and uPA inhibition. Regulation of basal levels of tPA in the neural tissues	Neuroplasticity Neuronal survival
PN-1	Neurons Astrocytes	Inhibition of uPA and thrombin	Neuroprotection
Plasminogen/Plasmin	Neurons Low levels in the brain tissues including the hippocampus, cortex, cerebellum and neuroendocrine tissues	Extracellular proteolysis	Recruitment of peripheral immune cells Microglia activation Neuroinflammation Hemostasis and vascular function Degradation of ECM components Long-term plasticity

**Table 2:** The PA system in animal models of epileptic activity.

PA System Component	Model/Object	PA System Role	References
TPA	PTZ-induced acute seizures/rat	Increase in mRNA expression in the hippocampus and cortex	[111,141]
	Electric kindling/rat	Increase in mRNA expression in the hippocampus	[111]
	KA-induced seizures/WT mice	Increase in tPA protein and enzymatic activity in the hippocampus and amygdala	[139,143]
	KA-induced seizures/tPA−/− mice	Increased resistance to seizures and reduced neuronal damage in tPA deficient mice Decreased mossy fiber outgrowth and sprouting in the dentate gyrus of tPA deficient mice	[10,143,145,151]
	Lithium-pilocarpine model of SE/rat	Increase in mRNA expression in the hippocampus on day 1 and day 3 after SE	[146]
	Electrically induced SE/rat	Increase in mRNA expression in the hippocampus during acute and latent phase after SE	[147,148]
	Amygdala kindling/transgenic heterozygous T4 mice (tPA overexpression)	Overexpression of tPA lowers the threshold for electrically induced seizures but does not affect epileptogenesis	[142]
	PTZ-induced seizures/WT mice	Increase in mRNA expression in the hippocampus, cortex, and amygdala 3 h after seizures	[140]
	Acute organophosphate intoxication-induced seizures/rat	Increase in tPA protein level in the hippocampus and cortex on days 1–7 after intoxication and up to day 28 in the hippocampus	[149]
PAI-1	KA-induced seizures/WT mice	Increase in mRNA expression in the limbic system and cortex	[11]
	Pilocarpine SE model/WT mice	Increase in PAI-1 protein expression up to 3 days after SE	[152]
	PTZ-induced seizures/WT mice	Increase in mRNA expression in the anterior cortex 72 h after seizures	[140]
NEUROSERPIN	KA-induced seizures/WT mice	Increase in protein level in response to KA-induced seizures Administration of exogenous neuroserpin delays the progression of seizure activity	[143]
	KA-induced seizures/Nsp−/− mice	Increase in seizure severity and decrease in the latency to onset of seizures in neuroserpin-deficient mice	[151]
UPA/UPAR	KA-induced seizures/WT mice	Increase in uPA mRNA expression in the limbic system and cortex	[11]
	Electrically induced SE/rat	Increase in uPA protein level and enzymatic activity Increase in uPAR expression in hippocampal interneurons	[153,154,169]
	PTZ-induced seizures/WT mice	Increased uPA and uPAR mRNA expression in the hippocampus and cortex	[140]
	PTZ-induced seizures/uPAR−/− mice	Spontaneous seizure activity in uPAR−/− mice Increased sensitivity to PTZ-induced seizures in uPAR−/− mice	[155]
	KA-induced epileptogenesis/uPAR−/− mice	No changes in sensitivity to KA in uPAR−/− mice but higher severity of spontaneous seizures and neurodegeneration	[156]
	Lithium-pilocarpine model of SE/rat	Increase in uPAR mRNA expression up to 5 months after SE	[150]
PAI-2	KA-induced seizures/WT mice	Increase in PAI-2 mRNA expression in the cortex, hippocampus, and amygdala	[162]
NEXIN-1	mTBI/WT mice	Increase in PN-1 expression in the later post mTBI stages Increase in sensitivity to seizure-like activity after thrombin injection	[163]

KA: kainic acid; PTZ: pentylenetetrazol; SE: status epilepticus; WT: wild type; tPA: tissue-type plasminogen activator; uPA: urokinase-type plasminogen activator; uPAR: urokinase-type plasminogen activator receptor; PAI: plasminogen activator inhibitor; PN-1: protease nexin-1; mTBI: mild traumatic brain injury.

**Table 3:** Changes in the PA system in epilepsy patents.

Disorder	Reported Changes in the PA System	References
Drug-resistant epilepsy in children	Increase in PAI-1 plasma level	[177]
Focal chronic refractory epilepsy	Increase in tPA, uPA, uPAR, and PAI-1 mRNA and protein expression in surgical specimens from patients with focal epilepsy	[178]
Intractable frontal lobe epilepsy	Increase in uPAR expression in frontal lobe	[179]
TLE	Increased plasma uPAR level, reversed after the removal of epileptogenic lesion Significant differences in genotypic and allelic frequencies of polymorphic sites of tPA gene *PLAT* between TLE patients and controls	[180,181]
Idiopathic epilepsy in children	Increase in serum tPA levels	[182]
FENIB	Polymerization of mutated neuroserpin leading to neurodegeneration and progressive myoclonus epilepsy	[192,193]

TLE: temporal lobe epilepsy; FENIB: familial encephalopathy with neuroserpin inclusion bodies; tPA: tissue-type plasminogen activator; uPA: urokinase-type plasminogen activator; uPAR: urokinase-type plasminogen activator receptor; PAI-1: plasminogen activator inhibitor 1.

**Table 4:** Changes in PA system reported in patients with depression, anxiety, and PTSD and in experimental models.

Disorder	Model(Condition)/Object	PA System Changes	References
Depression	Depression and depressive neurosis/patients	Decrease in tPA plasma levels, Decrease in plasma levels of total PAI-1 and tPA-PAI-1 complex	[253]
		Decrease in serum tPA and BDNF, decrease in proBDNF, reverse after antidepressant treatment	[15,255]
	MDD/patients	Increase in PAI-1 plasma level and mRNA expression	[256,257]
	MDD/geriatric patients	Decrease in PAI-1 plasma levels	[259]
	MDD/patients	rs2227684-G and rs7242-T alleles of the *SERPINE1* gene encoding PAI-1 associated with MDD	[260]
	MDD/human brain tissue	Increased PAI-1 expression in the hippocampal astrocytes of MDD patients	[257]
	LPS-induced depression model/WT mice	Increased PAI-1 plasma levels and expression in hippocampus	[257]
	PAI-1^−/−^ mice	PAI-1 deficiency led to the development of depressive-like phenotype with resistance to antidepressants	[261]
	First-episode depression/patients	Neuroserpin downregulation was found in the peripheral blood mononuclear cells	[262]
	Depressive syndrome during pregnancy/patients	Increase in neuroserpin plasma levels	[263]
	LPS-induced depression/rat	Decrease in mRNA expression of neuroserpin in the hippocampus and prefrontal cortex	[262]
Anxiety and stress	Anxiety disorders (panic disorder with agoraphobia or social phobia)/patients	Increase in plasma PAI-1 levels	[268]
	Panic disorder/patients	No changes in PAI-1 and tPA serum levels	[255]
	Restraint stress/WT mice	Increase in tPA expression and activity in the amygdala Increase in PAI-1 expression	[273]
	Restraint stress/tPA^−/−^ mice	Anxiety-like behavior in response to stress is reduced compared to WT mice	[273]
	tPA^null^ mice	Increased locomotor hyperactivity and reduced anxiety	[16]
PTSD	PTSD-like fear memory/ WT mice	Increased PAI-1 expression in response to corticosterone + fear conditioning	[14]
	VGV transgenic mice (5-HT2CR overexpression)	Decreased tPA and BDNF mRNA expression in the hippocampus, increased in the amygdala	[280]
	PTSD/pediatric patients	Decreased serum levels of BDNF and proBDNF, increased serum tPA levels	[281]
	PTSD/patients	Increased serum levels of tPA	[282]

MDD—major depressive disorder; PTSD—posttraumatic stress disorder; LPS—lipopolysaccharide; WT—wild type; BDNF—brain-derived neurotrophic factor; tPA—tissue-type plasminogen activator; PAI—plasminogen activator inhibitor; 5-HT2CR—serotonin 2C receptor.

## Data Availability

Data sharing is not applicable.

## Abbreviations

The following abbreviations are used in this manuscript:

AED	Antiepileptic Drug
BBB	Blood–brain barrier
BDNF	Brain-Derived Neurotrophic Factor
CNS	Central Nervous System
CSF	Cerebrospinal Fluid
COX-2	Cyclooxygenase 2
ECM	Extracellular Matrix
Erk	Extracellular signal-regulated kinase
FENIB	Familial Encephalopathy with Neuroserpin Inclusion Bodies
GFD	Growth Factor-like Domain
IL-1β	Interleukin 1 beta
iNOS	Inducible nitric oxide synthase
KA	Kainic Acid
LPS	Lipopolysaccharide
LTP	Long-Term Potentiation
mBDNF	Mature Brain-Derived Neurotrophic Factor
MDD	Major Depressive Disorder
mTBI	Mild Traumatic Brain Injury
NF-kB	Nuclear factor kB
NMDA	N-Methyl-D-Aspartate
PA	Plasminogen Activation/Plasminogen Activator
PAI-1	Plasminogen Activator Inhibitor-1
PAI-2	Plasminogen Activator Inhibitor-2
PN-1	Protease Nexin-1
proBDNF	Precursor form of Brain-Derived Neurotrophic Factor
pro-uPA	single-chain zymogen form of uPA
PTSD	Post-Traumatic Stress Disorder
PTZ	Pentylenetetrazol
RCL	Reactive Center Loop
SE	Status Epilepticus
tPA	Tissue-type Plasminogen Activator
TBI	Traumatic Brain Injury
TGF-β	Transforming growth factor beta
TLE	Temporal Lobe Epilepsy
TNF-α	Tumor Necrosis Factor-alpha
TLR	Toll-like receptor
Trk	Tropomyosin receptor kinase
uPA	Urokinase-type Plasminogen Activator
uPAR	Urokinase-type Plasminogen Activator Receptor
WT	Wild Type
